# Potential roles of mediator Complex Subunit 13 in Cardiac Diseases

**DOI:** 10.7150/ijbs.52290

**Published:** 2021-01-01

**Authors:** Wenqian Zhou, He Cai, Jia Li, He Xu, Xiang Wang, Hongbo Men, Yang Zheng, Lu Cai

**Affiliations:** 1 The Center of Cardiovascular Diseases, the First Hospital of Jilin University, Changchun 130021, China.; 2 Pediatric Research Institute, the Department of Pediatrics of University of Louisville, Louisville, KY 40202, USA.; 3 Department of Nephrology, the First Hospital of Jilin University, Changchun 130021, China.; 4 Department of Respiratory Medicine, the First Hospital of Jilin University (Eastern Division), Changchun 130031, China.; 5 Department of Pharmacology and Toxicology, the University of Louisville, Louisville, KY 40202, USA.

**Keywords:** MED13, cardiovascular diseases, energy metabolism

## Abstract

Mediator complex subunit 13 (MED13, previously known as THRAP1 and TRAP240) is a subunit of the cyclin-dependent kinase 8 (CDK8) kinase module in the eukaryotic mediator complex. MED13 has been known to play critical roles in cell cycle, development, and growth. The purpose of this review is to comprehensively discuss its newly identified potential roles in myocardial energy metabolism and non-metabolic cardiovascular diseases. Evidence indicates that cardiac MED13 mainly participates in the regulation of nuclear receptor signaling, which drives the transcription of genes involved in modulating cardiac and systemic energy homeostasis. MED13 is also associated with several pathological conditions, such as metabolic syndrome and thyroid disease-associated heart failure. Therefore, MED13 constitutes a potential therapeutic target for the regulation of metabolic disorders and other cardiovascular diseases.

## Introduction

Aberrant gene transcription causes serious human diseases 1. The mediator complex (MED) acts as a structural and functional bridge between DNA-binding transcription factors (TFs) and the basal transcriptional machinery 2. It helps regulate the expression of RNA polymerase II-transcribed genes, as well as transcription initiation and elongation 3. Mediator subunit 13 (MED13, known as THRAP1 and TRAP240) is part of the MED 4. MED13 maintains the transcription of some essential proteins involved in growth and development, and MED13 gene mutations are associated with various diseases, including cardiac diseases. MED13 plays critical roles in diseases like metabolic syndrome and cardiovascular disease. This review will cover the basic structure and functions of MED13 and its potential roles in metabolic and non-metabolic cardiac diseases. Understanding MED13 biology is critical for its optimal manipulation as a potential therapeutic target.

## MED and MED13 structure and function

**Figure 1:**
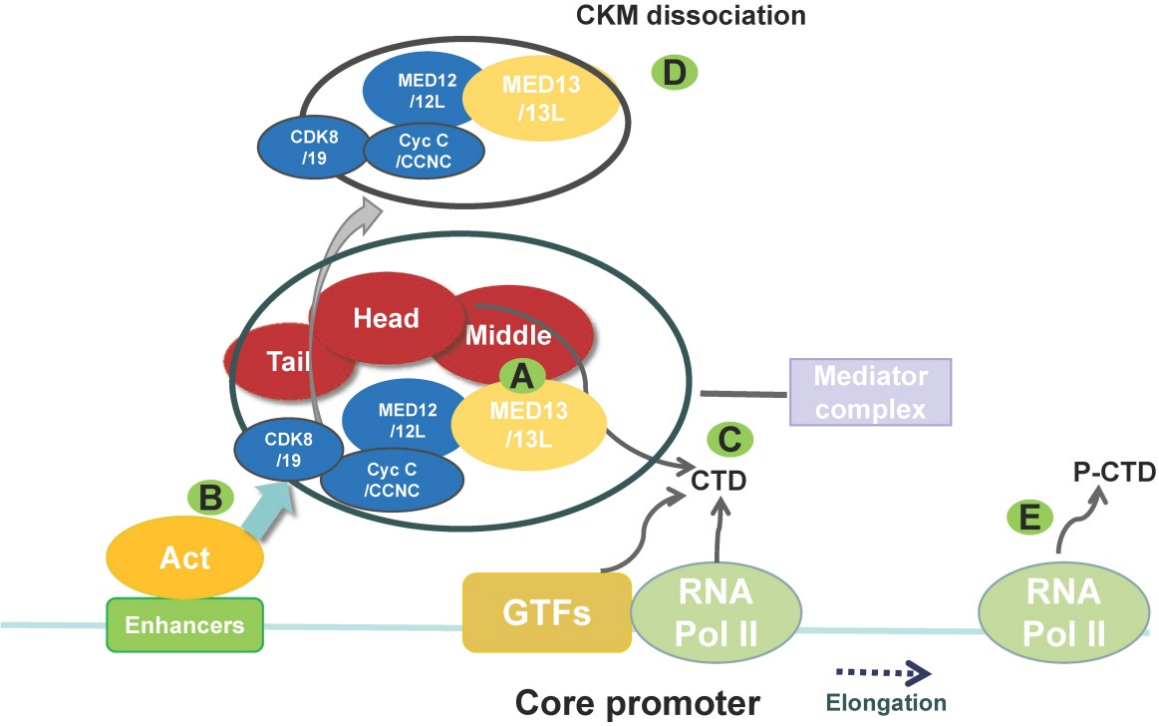
** The main structure and function of the mediator complex (MED).** The MED is composed of four parts: the head, middle, tail, and CKM. The CKM includes CDK8/19, Cyc C/CCNC, MED12/12L, and MED13/13L. (A) The CKM associates with the MED via interaction between MED13 and a “hook” at the end of the MED (middle module). Targeted degradation of MED13 can affect the association between the CKM and the MED. (B) An activator (Act) recruits the MED to an enhancer and then recruits GTFs. (C) The MED helps to recruit RNA polymerase (Pol) II to the promoter via interaction with the non-phosphorylated carboxy-terminal domain (CTD) of RNA polymerase II. MED also helps recruit other factors to promote the formation of the preinitiation complex. (D) This process includes CKM dissociation from the MED. (E) Then, the CTD of RNA polymerase II is phosphorylated (P-CTD) by the GTF TFIIH, which is accompanied by MED dissociation, thereby allowing RNA polymerase II to escape from the promoter and initiate transcription. The MED also regulates RNA polymerase II pausing and elongation. (CKM, cyclin-dependent kinase 8 (CDK8) kinase module; GTF, general transcription factor).

Eukaryotic transcription requires RNA polymerase II and various transcriptional repressors and activators, including TFs, that bind to specific DNA sequences in gene enhancer regions. TFs function is regulated by transcriptional co-regulators 5. The MED acts as a co-regulator and contains multiple subunits (approximately 25 and 30 subunits in yeast and humans, respectively). It functions as an adaptor between TFs bound to upstream regulatory elements and the transcription machinery, which includes RNA polymerase II and general TFs (GTFs), such as transcription initiation factor II (TFII) A, TFIIB, TFIID, TFIIE, TFIIF, and TFIIH 5-7. The MED is recruited by TFs to regulatory regions and binds to RNA polymerase II and GTFs that have been recruited to the core promoter to drive formation of the preinitiation complex. The MED mainly transmits signals from TFs to RNA polymerase II (**Figure 1**) and regulates the transcription of specific genes, such as those for nuclear receptors (NRs) 8, 9.

Electron microscopy data indicate that the MED is composed of four modules: the head, middle, tail, and cyclin-dependent kinase 8 (CDK8) kinase module (CKM) 5, 10. The CKM comprises CDK8, cyclin C (Cyc C/CCNC), MED13, and MED12 11 (**Figure 1**). Three CKM proteins, CDK8, MED13, and MED12, have paralogs that can replace them in the kinase domain, although they do not perform exactly the same functions 12: cyclin-dependent kinase 19, mediator subunit 13 like protein (MED13L, originally called PROSIT240 or THRAP2), and mediator subunit 12 like protein (MED12L). MED13L is similar in size to MED13, with which it shares 50% identity at the protein level 13. CKM can regulate transcription by blocking the MED-RNA polymerase II association, thereby preventing transcription initiation or re-initiation 14. The MED undergoes a conformational change and forms a dynamic bridge between enhancers and promoters via reversible dissociation of CKM from the MED 11, 15 (**Figure 1**).

MED13 plays a connective role between the CKM and the MED core 16; therefore, targeting MED13 may affect MED transcriptional co-regulatory function. In yeast, *Caenorhabditis elegans*, zebrafish, *Drosophila*, and mouse, the MED13 sequence is conserved, and the protein regulates gene expression in numerous physiological growth and developmental processes 12, 17-21 (Table 1). Moreover, MED13 is critical in myogenesis, zygotic genome activation, prevention of mitochondrial fission, and programmed cell death 12, 21, 22. In mice, MED13/MED13L has an essential function in embryonic development. Snijders Blok et al. 23 reported three missense mutations (p.Pro327Ser, p.Thr326Ile, and p.Pro327Gln) and one in-frame deletion (p.Thr326del) in a conserved phosphodegron of human MED13. Normally, this phosphodegron is recognized by the S-phase kinase-associated protein (Skp)-Cullin-F-box (SCF) F-box and WD repeat domain-containing 7 (Fbw7) ubiquitin ligase complex (SCF^Fbw7^), resulting in MED13 protein ubiquitination and degradation 24, suggesting that these mutations may affect MED13 protein turnover. Some MED13 gene mutations are reportedly associated with abnormal heart development 25, 26, neurodevelopmental disorders, nervous system disorders, craniofacial dysmorphisms, and muscle diseases 23, 27, 28 (**Table 1**). MED13L gene mutations are also associated with these phenotypes, but with more variable features 13, 26, 29-36. MED13 not only plays a critical role in necessary physiological processes, including organismal growth and development, but is also involved in pathophysiological processes and diseases.

## MED13 degradation under oxidative stress

Elevated levels of reactive oxygen species can be observed during aging and after exposure to environmental stress 22. In yeast, in response to oxidative stress, Cyc C is released from the nucleus into the cytoplasm, where it interacts with mitochondria to regulate cell death 44, 45. This process is dependent on MED13 degradation. Under normal conditions, MED13 is required for Cyc C retention in the nucleus, binding Cyc C via intrinsic disordered regions (IDRs) 22, 46. SCF protein GRR1 ubiquitin ligase complex (SCF^Grr1^)-mediated degradation of MED13 targets its IDRs. CDK8 phosphorylates MED13 in the absence of stress. However, in response to oxidative stress, the cell wall integrity/MAP kinase/Slt2 pathway is activated and Slt2 phosphorylates Cyc C and MED13. The phosphorylated sites are within the same MED13 IDRs that are associated with Cyc C and SCF^Grr1^ activity. CDK8- and Slt2-mediated phosphorylation of IDRs in MED13 release Cyc C from MED13, leading to its recognition by SCF^Grr1^ and subsequent degradation 47. In addition, the AMP kinase Snf1 activates another SCF^Grr1^-responsive degron of MED13 48. Thus, MED13 degradation plays a critical role in mediating Cyc C involvement in mitochondrial fragmentation and cell death following oxidative stress. However, in mammalian cells, only 10% of total Cyc C is released, and the overall level of Cyc C protein expression does not change under oxidative stress 46. In human cells, SCF^Fbw7^ regulates MED13 degradation and ubiquitylation, which may use a similar two-kinase mechanism of MED13 degradation 24, 47. Additional research investigating changes in MED13 during oxidative stress in higher eukaryotes is warranted.

## Pathophysiological role of MED13 in the heart

MED13 is ubiquitous in human tissues, with highest MED13 expression found in the placenta, skeletal muscle, heart, pancreas, and brain 49. MED13/MED13L plays an important role in physiological heart development. Pediatric patients with cyanotic congenital heart disease exhibit higher rates of adenosine-to-inosine (A-to-I) RNA editing of MED13 intronic segments than healthy individuals, which likely affects gene expression by altering RNA splicing and mRNA nuclear retention, among other mechanisms 25, 50. Moreover, defects in MED13L are related to transposition of the great arteries and coarctation of the aorta 13, 26, 33. Mutations of the human MED13L gene lead to MED13L haploinsufficiency syndrome, which includes congenital heart defects 40, 51. Cardiac MED13 is involved in systemic energy metabolism, and is associated with obesity, diabetes, and other diseases linked to energy metabolism. In addition, cardiac MED13 participates in regulation of cardiac function with thyroid hormone (TH).

### Cardiac MED13 modulates energy metabolism through NR signaling

Cardiac overexpression of MED13 in transgenic (α*-*myosin heavy chain [αMHC]-MED13-TG or MED13-cTG) mice promotes sensitivity to insulin and increases energy expenditure, thereby preventing high-fat diet (HFD)-induced obesity. Conversely, cardiac-specific deletion of MED13 promotes obesity in response to HFD and leads to metabolic syndrome, characterized by glucose intolerance and fatty liver 42. Moreover, the expression of cardiac MED13 mRNA is lower in Zucker diabetic fatty rats than in their lean counterparts 52. This finding is consistent with the function of cardiac MED13 in metabolic regulation. In addition, the expression of cardiac MED13 mRNA in female Zucker lean rats is higher than that in males, which may explain why female rats are able to maintain insulin sensitivity and glucose tolerance while male rats are not. This sex-specific difference in MED13 expression indicates that MED13 is a protective factor in the hearts of healthy female rats 52.

**Figure 2:**
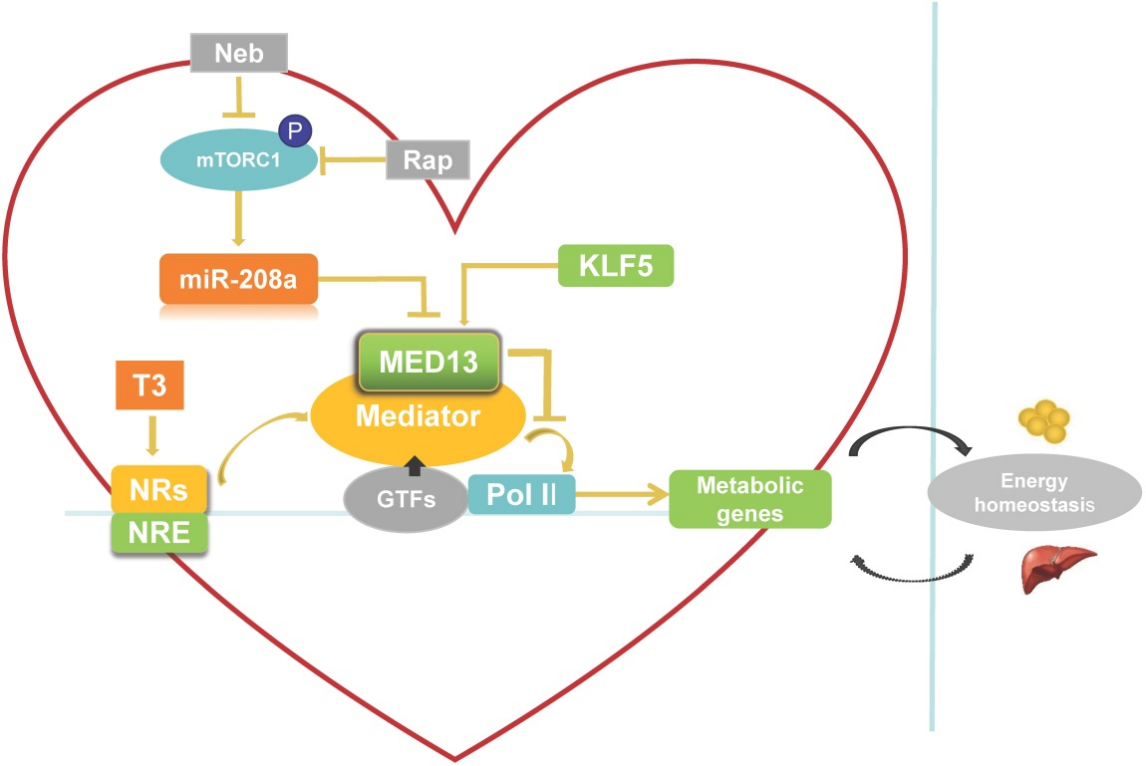
** Role of cardiac-specific MED13 in systemic energy metabolism.** Nebivolol (Neb) and rapamycin (Rap) can inhibit expression of miR-208a by suppressing activation of mTORC1. The expression of MED13, a downstream target gene of miR-208a, is downregulated by miR-208a. Overexpression of cardiac MED13 inhibits the association of RNA polymerase II (Pol II) with the MED, thereby repressing transcription and expression of NR target genes involved in cardiac energy metabolism. Changes in cardiac energy metabolism alter systemic energy metabolism. Another regulator of cardiac MED13 is KLF5, which can promote MED13 expression in the heart. (MED, mediator complex; NRs, nuclear receptors; NRE, NR response element; T3, triiodothyronine).

The heart requires a constant supply of energy, which is maintained by communication between the heart and energy storage depots in the body. Thus, heart energy metabolism affects whole-body energy metabolism and homeostasis (**Figure 2**). Expression of cardiac energy metabolism genes can be regulated by NRs 53. The MED reportedly interacts with NRs and NR response element (NRE) as a transcriptional co-activator when triiodothyronine (T3, a thyroid hormone) is present 54. MED13 in the heart inhibits MED-dependent NR signaling 42 to suppress the transcription of some NR-responsive metabolic genes (**Figure 2**). Deletion of these downregulated NR-responsive genes can result in resistance to HFD-induced obesity and protection against insulin resistance 42. In addition, other TFs involved in regulation of lipid biosynthesis and metabolism, such as NCoR, SREBP, and PPARγ 53, 55, 56, can be modulated by cardiac MED13 as responsive genes 42. Therefore, cardiac overexpression of MED13 represses metabolic genes in the heart to drive whole-body energy metabolism.

### Signaling pathways that potentially regulate cardiac MED13 expression

There are two potential pathways that regulate cardiac MED13 involvement in energy metabolism: the mammalian target of rapamycin complex 1 (mTORC1)/miR-208a/MED13 pathway and the Krüppel-like transcription factor 5 (KLF5)/MED13 pathway. Regarding the first potential pathway, chronic over-nutrition can lead to excessive activation of mTORC1 and downstream signaling, thereby inducing metabolic disorder 57. Therefore, increased mTORC1 activation is a key initiator of obesity 57, 58. Rapamycin is a canonical inhibitor of mTORC1. Nebivolol can also suppress mTORC1 activation in the hearts of Zucker obese rats 59. Gul et al. 60 demonstrated that rapamycin and nebivolol, which confer resistance to obesity in animal models, suppress mTORC1 activation and miR-208a expression in HL-1 cardiomyocytes. These results suggest that mTORC1 promotes the expression of miR-208a (**Figure 2**), a cardiac-specific small noncoding RNA encoded by an intron in the α-MHC gene. Deletion of miR-208a gene can increase MED13 expression, suggesting that miR-208a is negatively regulating MED13 expression 61 (**Figure 2**). Conversely, upregulating MED13 expression confers resistance to obesity in animal models upon pharmacologic miR-208a inhibition or rapamycin treatment. These results suggest that miR-208a is an upstream target of MED13, and reports have confirmed a miR-208a binding target site in the 3′ untranslated region (UTR) of the MED13 gene 61-63. Taken together, these findings suggest that the mTORC1/miR-208a/MED13 axis plays a role in regulating cardiac and systemic energy metabolism. In agreement with these results, exercise training can prevent obesity and cardiac pathological hypertrophy by increasing MED13 expression via regulation of miR-208a 64.

The second potential pathway is the KLF5/MED13 signaling pathway. KLF5 as one of the KLF family is a TF to involve in regulating lipid metabolism 65. KLF5 in the heart reportedly modulates the expression of cardiac fatty acid metabolism-related genes 66, 67. Moreover, deletion of cardiac KLF5 results in susceptibility to diet-induced obesity in mice, which can be suppressed by expression of cardiac MED13. Cardiac MED13 expression decreases in αMHC-KLF5^-/-^ mice. Furthermore, KLF5 overexpression increases MED13 expression in cardiomyocytes 68 (**Figure 2**). Thus, MED13 may be the key downstream target of KLF5 for metabolic regulation. Using promoter analysis, two KLF5-binding sites were identified in mouse and human MED13 promoters. Furthermore, chromatin immunoprecipitation experiments with HL-1 cells suggest the presence of a KLF5-binding site in the mouse MED13 promoter 68. These results indicate that KLF5 is a positive regulator of cardiac MED13 mediated metabolic regulation.

### Coordination of cardiac MED13 and thyroid hormone to regulate cardiac transcription

**Figure 3:**
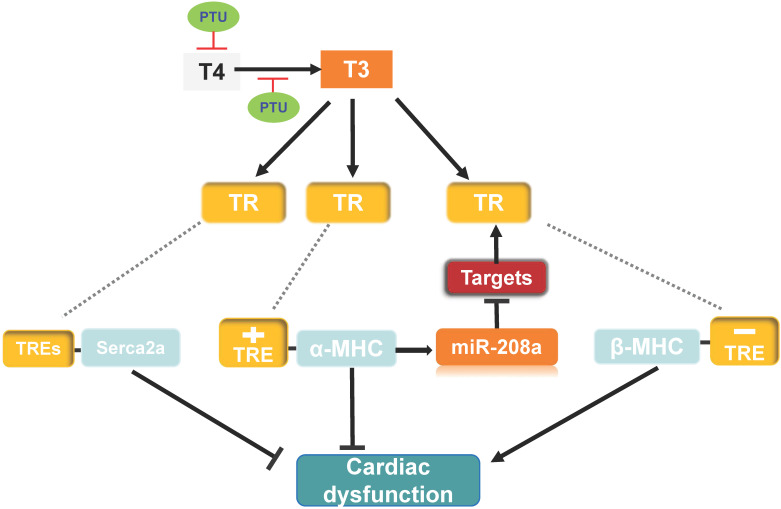

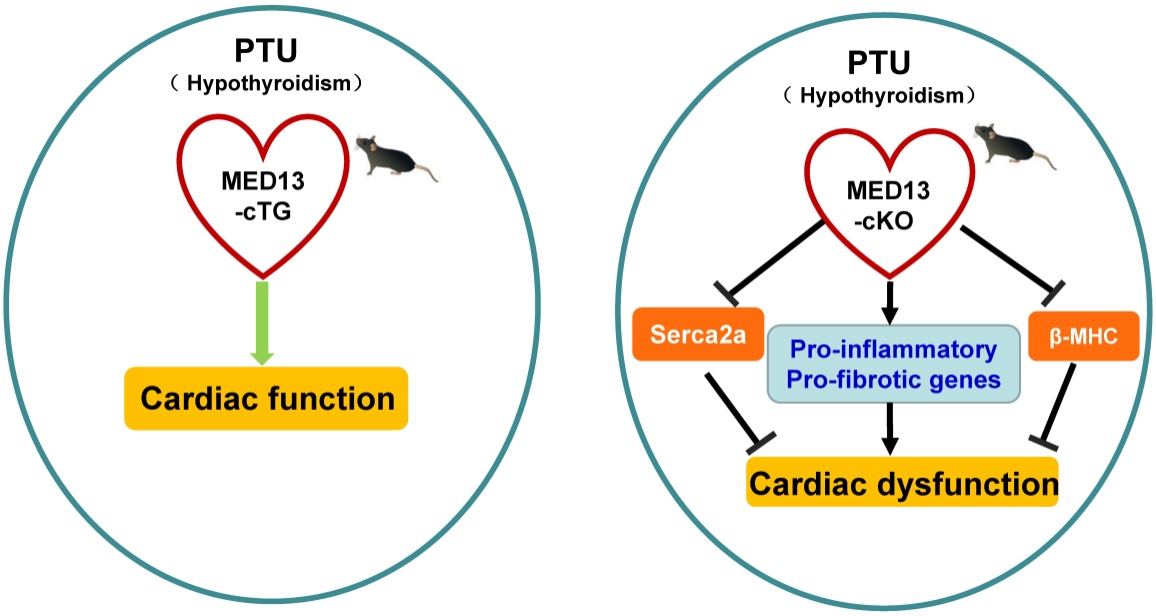
** Possible mechanism of MED13-mediated regulation of cardiac function in response to hypothyroidism. Top panel:** The T3 induces α-MHC gene transcription through TR binding a positive TRE and inhibits β-MHC transcription via TR binding a negative TRE. The intron in the α-MHC gene encodes miR-208a. In the context of hypothyroidism (PTU inhibits T4 synthesis and suppresses conversion from T4 to T3), miR-208a may inhibit cardiac MED13 and additional targets expression, which regulates TH receptor activity and β-MHC gene expression. T3 also enhances expression of Serca2a via TR binding TREs in its regulatory region. Changes in the level of α/β-MHC and Serca2a expression could affect cardiac function in the context of hypothyroidism. **Lower panel:** Cardiac contractility function is suppressed by PTU. Overexpression of cardiac MED13 ameliorates cardiac dysfunction induced by hypothyroidism, whereas deletion of cardiac MED13 aggravates hypothyroidism-related cardiac dysfunction. The expression of serca2a and myh7 are lower in the hearts of MED13-cKO mice than wild-type mice treated with PTU and pro-inflammatory/pro-fibrotic genes expression are higher in MED13-cKO mice. These changes are associated with worse cardiac dysfunction in MED13-cKO mice than in wild-type mice. (PTU, propylthiouracil; T4, thyroxine; T3, triiodothyronine; TR, thryoid hormone receptor; TRE, thyroid hormone response element).

There is a close relationship between thyroid hormone [TH, including T3 and thyroxine (T4)] and cardiovascular system 69-71. High level of TH increases heart rate, cardiac output, and myocardial contractility. Decreased TH causes opposite functions 71. Clinical and animals evidence indicate that hypothyroidism can induce heart failure and is associated with poor prognosis 72-74. The main role of TH in the heart is to regulate cardiac-specific gene transcription by interacting with TH receptors (TRs), and TRs bind to thyroid hormone response elements (TREs) in gene promoter regions 71. Cardiac contractility function can be affected by expressions of the α-MHC (*myh6*) and β-MHC (*myh7*) genes. TH stimulates expression of the α-MHC gene and inhibits expression of the β-MHC gene in the heart after birth 75 (**Figure 3**). Conversely, hypothyroidism suppress α-MHC and induce β-MHC expression, which is associated with cardiac dysfunction 63, 71. Although β-MHC is the main isoform in the ventricles of healthy adult humans, even small changes in expression of the two isoforms can meaningfully alter cardiac function. TH also positively regulates sarcoplasmic reticulum Ca^2+^ ATPase (*Serca2a*) gene expression 69, 76, 77 (**Figure 3**). Serca2a is an important Ca^2+^ pump for the maintenance of Ca^2+^ homeostasis, which is associated with myocardial calcium handling and plays a critical role in maintaining cardiac function 78, 79.

To investigate whether miR-208a is required for hypothyroidism-up-regulating β-MHC, van Rooij et al. treat miR-208a^-/-^ mice with propylthiouracil (PTU, an inhibitor of T3) to make a hypothyroidism model, and found that β-MHC is not upregulated in response to hypothyroidism 63, suggesting that miR-208a is required for increased expression of β-MHC by hypothyroidism. MED13 (known as thyroid hormone receptor associated protein 1), a downstream target of miR-208a 63, can regulate transcription and the activity of TRs via RNA polymerase II and GTF recruitment 42, 63, 80, 81. Therefore, in response to hypothyroidism, there would be prediction that overexpression of miR-208a inhibits MED13 and additional targets expression, thereby enhancing β-MHC expression via reducing the repressive activity of TRs towards β-MHC and altering cardiac function (**Figure 3**).

To further test the impact of cardiac MED13 on the response of the heart to low level of TH (hypothyroidism), Olson et al. treated MED13-cTG mice with PTU, which displayed increased cardiac systolic function compared with wild-type mice 42 (**Figure 3**). Moreover, in the context of hypothyroidism, deletion of cardiac MED13 exacerbates cardiac dysfunction. The expressions of *myh7* and *Serca2a* genes are lower in cardiac MED13 knockout (MED13-cKO) mice than wild-type mice 82, suggesting that cardiac MED13 plays a role in cardiac function conservation in hypothyroidism, as *myh7* and* Serca2a* expressions are necessary to maintain normal cardiac function.

The expression cardiac MED13 is increased in response to hypothyroidism and the increased MED13 expression might repress cardiac pro-inflammatory and pro-fibrotic gene transcriptions 82, which may be a potential mechanism for MED13 modulating cardiac transcription in the context of hypothyroidism (**Figure 3**).

### Involvement of MED13 in non-metabolic cardiovascular diseases

Cardiac MED13 expression is mainly involved in energy homeostasis. However, whether the effects of MED13 on cardiac energy metabolism influence cardiac function remains unclear. In addition, miR-208b-3p/MED13/Wnt signaling plays a role in cardiomyocyte apoptosis in the hypoxic/reoxygenated state 83. miR-208 is downregulated during right ventricular failure, which activates the MED13/NCoR1 pathway and inhibits myocyte enhancer factor 2 (MEF2) 84. Additional research is required to determine whether cardiac MED13 is involved in other cardiovascular diseases and if it regulates cardiac function.

## Potential MED13 crosstalk among organs

The interactions between metabolic organs and the central nervous system are crucial for maintaining whole-body energy homeostasis and may be altered in metabolic diseases such as obesity and diabetes. The central nervous system and peripheral organs produce bioactive molecules to communicate with other tissues, including cytokines and hormones 85. Cardiac and skeletal muscles are the key organs involved in regulating energy metabolism. MED13 is expressed in multiple organs associated with energy metabolism that participate in crosstalk.

### Heart-adipose tissue/liver crosstalk

**Figure 4:**
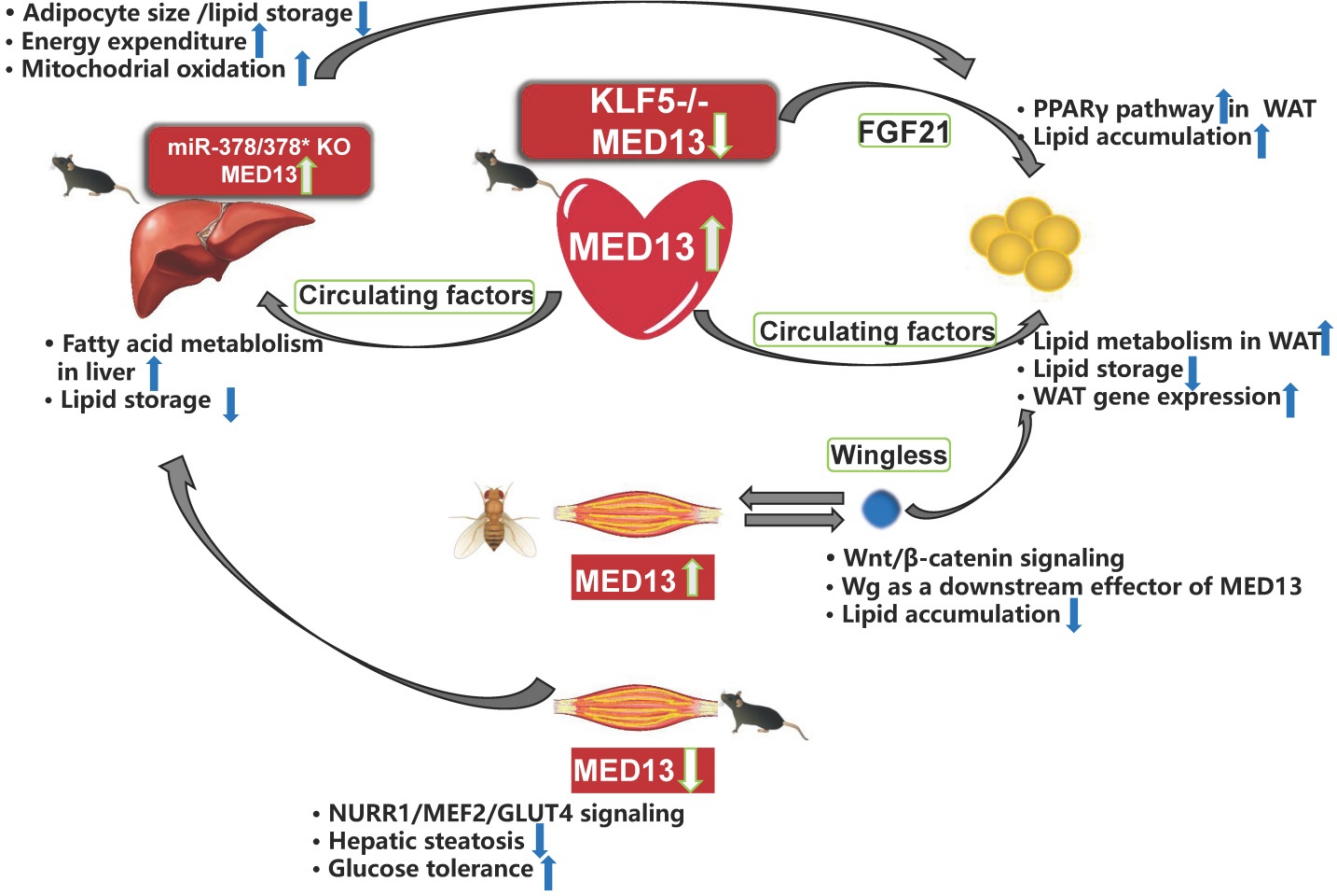
** Crosstalk among energy metabolism organs mediated by MED13.** Overexpression of cardiac MED13 in mice increases liver and WAT fatty acid metabolism and reduces lipid accumulation in these tissues via regulation of circulating factors. Genetic deletion of KLF5 in the mouse heart induces lower MED13 expression and higher FGF21 expression in the plasma and heart, which activates the PPARγ pathway in WAT and induces obesity. Expression of hepatic MED13 and mitochondrial fatty acid metabolism are higher in miR-378/378* knockout mice than in wild-type controls. However, opposite effects are observed upon overexpression of muscle-specific MED13 in mice and Drosophila. Upregulation of muscle MED13 in Drosophila activates the Wnt/β-catenin pathway in the muscle and adipose tissues. Wingless (Wg) acts downstream of muscle MED13 in Drosophila to enhance lipid metabolism and suppress obesity. In mice, genetic deletion of muscle MED13 improves glucose tolerance and metabolism, and confers resistance to hepatic steatosis. Moreover, skeletal muscle MED13 represses the NURR1/MEF2 cooperative pathway, thereby reducing the expression of the glucose-handling gene GLUT4 (WAT, white adipose tissue).

The heart requires a considerable energy supply, and may signal peripheral organs to convey its needs. Therefore, cardiac MED13, which affects cardiac energy metabolism, may control whole-body energy homeostasis. Olson et al. 42 reported that overexpression of MED13 in the mouse heart reduces fat accumulation in peripheral tissues. Deletion of cardiac MED13 in mice or *Drosophila* increases their susceptibility to HFD-induced obesity 42, 86. In addition, MED13-cTG mice exhibit 60% greater lipid clearance rate in the blood than that in wild-type mice 87. Lipid oxidation in the white adipose tissue (WAT) and liver is also higher in MED13-cTG mice than in wild-type mice. Furthermore, lipid uptake, fatty acid β-oxidation, mitochondrial content, energy consumption, and many genes involved in fatty acid metabolism and the Krebs cycle are upregulated in the WAT and liver of MED13-cTG mice, leading to a lean phenotype 88. The heart appears to send metabolic signals to peripheral tissues via circulating factors (**Figure 4**) because heterotypic wild-type parabiots have a lean phenotype in heterotypic parabiosis experiments 88. Several cardiokines, including atrial natriuretic factor and B-type natriuretic peptides, are involved in cardiac physiological and pathological processes 87. However, the serum levels of metabolic hormones and cardiokines are not elevated in MED13-cTG mice, and other circulating factors levels need to be confirmed 88. Moreover, in αMHC-KLF5^-/-^ mice, cardiac MED13 expression is lower than in wild-type mice, whereas the levels of FGF21 in the plasma and heart are higher in HFD-fed αMHC-KLF5^-/-^ mice. Circulating FGF21 activates the PPARγ pathway in the WAT and induces obesity 68 (**Figure 4**). However, it remains unknown if elevated FGF21 levels play a role in the effects of cardiac MED13 on obesity.

### Muscle-adipose tissue/liver crosstalk

Muscle plays a central role in systemic energy metabolism, and functions as a secretory organ. MED13 mediates crosstalk between skeletal muscle and adipose tissue of *Drosophila*, and between muscle and adipose tissue/liver of mice. Olson et al. 86 reported that expression of MED13 in muscle in *Drosophila* controls obesity. In addition, muscle-specific knockdown of MED13 in mice increases their susceptibility to obesity. Therefore, the function of MED13 in the muscle of Drosophila is the same as its role in the heart, with regard to adipose tissue crosstalk. The muscle-secreted obesity-associated factor Wingless (Wg) functions as a downstream target of MED13 to mediate muscle-adipose crosstalk and repress obesity 86 (**Figure 4**).

The effect of murine muscle MED13 on metabolic homeostasis is opposite to that of cardiac MED13. Deletion of muscle MED13 in mice (MED13-mKO) fed HFD improves their glucose tolerance, enhances muscle glucose metabolism, and prevents hepatic steatosis 89 (**Figure 4**). In addition, RNA sequencing data reveals that gene expressions of nuclear receptor NURR1, salt-inducible kinase 1 (which activates the TF MEF2), and glucose transporter type 4 (GLUT4) are upregulated in MED13-mKO muscle. Muscle-specific MED13 primarily represses the MEF2/NURR1 cooperative pathway, thereby suppressing expression of glucose handling genes, such as GLUT4 89-91. The opposite functions of MED13 in the heart and muscle in systemic energy metabolism suggest tissue-specific functions for MED13 via various signaling pathways.

### Liver-adipose tissue crosstalk

The liver is also an organ associated with energy metabolism, and MED13 mediates crosstalk between the liver and adipose tissue. The 3′ UTR of the MED13 gene includes three sites that can be recognized by sequences of miR-378*, and a luciferase reporter assay demonstrates repression of the MED13 3′ UTR in response to increasing expression of miR-378/miR-378* 43. In addition, mice with genetic deletion of miR-378/miR-378* exhibit higher hepatic MED13 expression than wild-type mice, and hepatic MED13 regulates energy homeostasis via the transcriptional network regulated by the transcriptional co-regulator PGC-1β 43, 50. The miR-378/miR-378* knockout mice display resistance to HFD-induced obesity, with less fat accumulation in the WAT than their wild-type counterparts 43 (**Figure 4**). These findings suggest that hepatic MED13 is a target of miR-378*, and that hepatic MED13 could influence adipose tissue and obesity.

Furthermore, WAT and brown adipose tissue can be interconverted through a process known as adipose organ remodeling. MED13 is expressed in various energy metabolism organs and mediates crosstalk via circulating factors or other signaling pathways. Therefore, MED13-mediated crosstalk may participate in adipose organ remodeling through endocrine factors 92.

## The role of MED12 in cardiac diseases and its link with MED13

MED12, as a part of CKM, is closely related to MED13. MED12 also plays a critical role in the development and growth of organisms just like MED13 19, 93-97. Reports have showed that mutation of the MED12 gene in zebrafish and mouse affected heart development 98, 99. In the MED12 gene mutant mouse embryonic stem (ES) cell model, embryos live no more than 10.5 days and exhibit heart malformations, including enlarged hearts and cardiac dysfunction. Furthermore, the canonical Wnt and Wnt/β-catenin signaling is impaired in this model 99. In addition, MED12 was reported to interact with Nanog to regulate Nanog target genes in mouse ES cells, but these results cannot be reproduced in other models 99, 100. An alternative transcript of the MED12 gene may be involved in endothelial differentiation 101. Reportedly mutations of the MED12 gene in humans cause similar phenotypes as mutations in the MED13 and MED13L genes. MED12-associated disorders include Opitz-Kaveggia syndrome (FG syndrome) type 1, Lujan syndrome, and X-linked Ohdo syndrome, and all that share cardiac malformations 102-106.

MED12 and MED13 have similar functions in controlling obesity in* Drosophila*, especially heart and muscle MED12 and MED13. Knockdown of cardiac MED12 and MED13 in *Drosophila* increases fat accumulation and induces obesity, which suggests that like MED13, cardiac MED12 also can control metabolism homeostasis. In addition, deletion of muscle MED12 and MED13 in *Drosophila* also results in similar obesity phenotypes 86.

## Conclusions and Perspective

MED13 regulates transcription and influences organismal growth and development 107. In humans, MED13 gene mutations are associated with abnormal organ development, leading to congenital heart defects and neurological disorders. To date, the functions of MED13 have been mainly characterized in cardiac and systemic energy metabolism, through which it is associated with metabolic diseases such as obesity and diabetes. Understanding the molecular mechanisms underlying MED13-associated disorders and pathological conditions will facilitate the identification of potential therapeutic targets for clinical application. For example, miR-208a and miR-378/378* reportedly downregulate MED13 expression. Therefore, pharmacological agents targeting these miRNAs, such as nebivolol and rapamycin, may prove therapeutic by modulating expression of cardiac MED13 or MED13-regulated cardiokines in metabolic disorders 60, 87.

Nevertheless, we must consider that these therapeutic interventions may cause unintended effects due to the diverse functions of MED13 in different tissues. miRNA antagonists or agonists will affect many targets and thus require thorough investigation for adverse effects. Dexmedetomidine has been shown to protect cardiomyocytes from apoptosis through the miR-208b-3p/MED13/Wnt signaling pathway under hypoxic conditions. Furthermore, MED13 regulates cardiac transcription with TH under hypothyroid conditions. In conclusion, MED13 provides a possible therapeutic target in metabolic and non-metabolic cardiac diseases. However, many challenges to their clinical application remains, in particular, the accompanying adverse effects need to be elucidated.

## Figures and Tables

**Table 1:** Comparison of the biological roles and associated phenotypes of MED13 and MED13L

	MED13	MED13L	References
Human chromosome	17q23.2	12q24.21	37
Biological role	Growth and development/Myogenesis	Neural crest induction/Neurogenesis	19-21, 38-40
	Zygotic genome activation/Mitosis/Mitochondrial fission/PCD	Cell proliferation	12, 17, 18, 22, 41
Pathophysiological role	Target of miR-208a and miR-378/378*	—	42, 43
	Cyanotic congenital heart disease (A-to-I RNA editing)	Congenital heart defect (TGA)/Coarctation of the aorta	13, 25, 33
	Neurodevelopmental disorder (DD/ID)	MED13L haploinsufficiency syndrome	23, 26, 29-32, 34, 40
	ASD/ADHD	(broaden the clinical features)	

PCD: Programmed cell death; DD: Developmental delay; ID: Intellectual disability; A-to-I RNA editing: adenosine-to-inosine RNA editing; ASD: Autism spectrum disorder; ADHD: Attention deficit hyperactivity disorder; TGA: Transposition of the great arteries.

## Abbreviations

MED13: Mediator complex subunit 13
MED13L: Mediator subunit 13 like protein
CDK8: Cyclin-dependent kinase 8
MED: Mediator complex
TFs: Transcription factors
GTFs: General transcription factors
TFII: Transcription initiation factor II
NRs: Nuclear receptors
CKM: Cyclin-dependent kinase 8 kinase module
Cyc C/CCNC: Cyclin C
MED12: Mediator complex subunit 12
MED12L: Mediator subunit 12 like protein
CDK19: Cyclin-dependent kinase 19
RNA Pol II: RNA polymerase II
SCF: S-phase kinase-associated protein-Cullin-F-box
Fbw7: F-box and WD repeat domain-containing 7
IDRs: Intrinsic disordered regions
A-to-I: Adenosine-to-inosine
TH: Thyroid hormone
HFD: High fat diet
mTORC1: Mammalian target of rapamycin complex 1
KLF5: Krüppel-like transcription factor 5
UTR: Untranslated region
TRs: Thyroid hormone receptors
TREs: Thyroid hormone-response elements
MHC: Myosin heavy chain
Serca2a: Sarcoplasmic reticulum Ca^2+^ ATPase
WAT: White adipose tissue
MEF2: Myocyte enhancer factor 2
ES cells: Embryonic stem cells

## References

[1] Dannappel MV, Sooraj D, Loh JJ, Firestein R (2018). Molecular and *in vivo* Functions of the CDK8 and CDK19 Kinase Modules. Front Cell Dev Biol.

[2] Borggrefe T, Yue X (2011). Interactions between subunits of the Mediator complex with gene-specific transcription factors. Semin Cell Dev Biol.

[3] Putlyaev EV, Ibragimov AN, Lebedeva LA, Georgiev PG, Shidlovskii YV (2018). Structure and Functions of the Mediator Complex. Biochemistry (Mosc).

[4] Poss ZC, Ebmeier CC, Taatjes DJ (2013). The Mediator complex and transcription regulation. Crit Rev Biochem Mol Biol.

[5] Soutourina J (2018). Transcription regulation by the Mediator complex. Nat Rev Mol Cell Biol.

[6] Roeder RG (2005). Transcriptional regulation and the role of diverse coactivators in animal cells. FEBS letters.

[7] Schiano C, Casamassimi A, Vietri MT, Rienzo M, Napoli C (2014). The roles of mediator complex in cardiovascular diseases. Biochim Biophys Acta.

[8] Allen BL, Taatjes DJ (2015). The Mediator complex: a central integrator of transcription. Nat Rev Mol Cell Biol.

[9] Malik S, Roeder RG (2005). Dynamic regulation of pol II transcription by the mammalian Mediator complex. Trends Biochem Sci.

[10] Jeronimo C, Langelier MF, Bataille AR, Pascal JM, Pugh BF, Robert F (2016). Tail and Kinase Modules Differently Regulate Core Mediator Recruitment and Function *In vivo*. Mol Cell.

[11] Petrenko N, Jin Y, Wong KH, Struhl K (2016). Mediator Undergoes a Compositional Change during Transcriptional Activation. Mol Cell.

[12] Miao YL, Gambini A, Zhang Y, Padilla-Banks E, Jefferson WN, Bernhardt ML (2018). Mediator complex component MED13 regulates zygotic genome activation and is required for postimplantation development in the mouse. Biol Reprod.

[13] Muncke N, Jung C, Rüdiger H, Ulmer H, Roeth R, Hubert A (2003). Missense mutations and gene interruption in PROSIT240, a novel TRAP240-like gene, in patients with congenital heart defect (transposition of the great arteries). Circulation.

[14] Verger A, Monte D, Villeret V (2019). Twenty years of Mediator complex structural studies. Biochem Soc Trans.

[15] Jeronimo C, Robert F (2017). The Mediator Complex: At the Nexus of RNA Polymerase II Transcription. Trends Cell Biol.

[16] Fant CB, Taatjes DJ (2019). Regulatory functions of the Mediator kinases CDK8 and CDK19. Transcription.

[17] Banyai G, Lopez MD, Szilagyi Z, Gustafsson CM (2014). Mediator can regulate mitotic entry and direct periodic transcription in fission yeast. Mol Cell Biol.

[18] Yoda A, Kouike H, Okano H, Sawa H (2005). Components of the transcriptional Mediator complex are required for asymmetric cell division in C. elegans. Development.

[19] Treisman J (2001). Drosophila homologues of the transcriptional coactivation complex subunits TRAP240 and TRAP230 are required for identical processes in eye-antennal disc development. Development.

[20] Lin X, Rinaldo L, Fazly AF, Xu X (2007). Depletion of Med10 enhances Wnt and suppresses Nodal signaling during zebrafish embryogenesis. Dev Biol.

[21] Tripathi S, Miyake T, McDermott JC (2019). Smad7:β-catenin complex regulates myogenic gene transcription. Cell Death Dis.

[22] Khakhina S, Cooper KF, Strich R (2014). Med13p prevents mitochondrial fission and programmed cell death in yeast through nuclear retention of cyclin C. Mol Biol Cell.

[23] Snijders Blok L, Hiatt SM, Bowling KM, Prokop JW, Engel KL, Cochran JN (2018). De novo mutations in MED13, a component of the Mediator complex, are associated with a novel neurodevelopmental disorder. Hum Genet.

[24] Davis MA, Larimore EA, Fissel BM, Swanger J, Taatjes DJ, Clurman BE (2013). The SCF-Fbw7 ubiquitin ligase degrades MED13 and MED13L and regulates CDK8 module association with Mediator. Genes Dev.

[25] Borik S, Simon AJ, Nevo-Caspi Y, Mishali D, Amariglio N, Rechavi G (2011). Increased RNA editing in children with cyanotic congenital heart disease. Intensive Care Med.

[26] Asadollahi R, Oneda B, Sheth F, Azzarello-Burri S, Baldinger R, Joset P (2013). Dosage changes of MED13L further delineate its role in congenital heart defects and intellectual disability. Eur J Hum Genet.

[27] Boutry-Kryza N, Labalme A, Till M, Schluth-Bolard C, Langue J, Turleau C (2012). An 800 kb deletion at 17q23.2 including the MED13 (THRAP1) gene, revealed by aCGH in a patient with a SMC 17p. Am J Med Genet A.

[28] Nowak R, Szota J, Mazurek U (2012). Vitamin D receptor gene (VDR) transcripts in bone, cartilage, muscles and blood and microarray analysis of vitamin D responsive genes expression in paravertebral muscles of juvenile and adolescent idiopathic scoliosis patients. BMC Musculoskelet Disord.

[29] van Haelst MM, Monroe GR, Duran K, van Binsbergen E, Breur JM, Giltay JC (2015). Further confirmation of the MED13L haploinsufficiency syndrome. Eur J Hum Genet.

[30] Adegbola A, Musante L, Callewaert B, Maciel P, Hu H, Isidor B (2015). Redefining the MED13L syndrome. Eur J Hum Genet.

[31] Cafiero C, Marangi G, Orteschi D, Ali M, Asaro A, Ponzi E (2015). Novel de novo heterozygous loss-of-function variants in MED13L and further delineation of the MED13L haploinsufficiency syndrome. Eur J Hum Genet.

[32] Smol T, Petit F, Piton A, Keren B, Sanlaville D, Afenjar A (2018). MED13L-related intellectual disability: involvement of missense variants and delineation of the phenotype. Neurogenetics.

[33] Chen CP, Chen YY, Chern SR, Wu PS, Su JW, Chen YT (2013). Prenatal diagnosis and molecular cytogenetic characterization of de novo partial trisomy 12q (12q24.21->qter) and partial monosomy 6q (6q27->qter) associated with coarctation of the aorta, ventriculomegaly and thickened nuchal fold. Gene.

[34] Torring PM, Larsen MJ, Brasch-Andersen C, Krogh LN, Kibaek M, Laulund L (2019). Is MED13L-related intellectual disability a recognizable syndrome?. Eur J Med Genet.

[35] Poot M (2020). Mutations in Mediator Complex Genes CDK8, MED12, MED13, and MEDL13 Mediate Overlapping Developmental Syndromes. Mol Syndromol.

[36] Calpena E, Hervieu A, Kaserer T, Swagemakers SMA, Goos JAC, Popoola O (2019). De Novo Missense Substitutions in the Gene Encoding CDK8, a Regulator of the Mediator Complex, Cause a Syndromic Developmental Disorder. Am J Hum Genet.

[37] Napoli C, Sessa M, Infante T, Casamassimi A (2012). Unraveling framework of the ancestral Mediator complex in human diseases. Biochimie.

[38] Xu M, Chen X, Chen D, Yu B, Li M, He J (2018). MicroRNA-499-5p regulates skeletal myofiber specification via NFATc1/MEF2C pathway and Thrap1/MEF2C axis. Life Sci.

[39] Utami KH, Winata CL, Hillmer AM, Aksoy I, Long HT, Liany H (2014). Impaired development of neural-crest cell-derived organs and intellectual disability caused by MED13L haploinsufficiency. Hum Mutat.

[40] Asadollahi R, Zweier M, Gogoll L, Schiffmann R, Sticht H, Steindl K (2017). Genotype-phenotype evaluation of MED13L defects in the light of a novel truncating and a recurrent missense mutation. Eur J Med Genet.

[41] Angus SP, Nevins JR (2012). A role for Mediator complex subunit MED13L in Rb/E2F-induced growth arrest. Oncogene.

[42] Grueter CE, van Rooij E, Johnson BA, DeLeon SM, Sutherland LB, Qi X (2012). A cardiac microRNA governs systemic energy homeostasis by regulation of MED13. Cell.

[43] Carrer M, Liu N, Grueter CE, Williams AH, Frisard MI, Hulver MW (2012). Control of mitochondrial metabolism and systemic energy homeostasis by microRNAs 378 and 378*. Proc Natl Acad Sci U S A.

[44] Strich R, Cooper KF (2014). The dual role of cyclin C connects stress regulated gene expression to mitochondrial dynamics. Microb Cell.

[45] Ježek J, Smethurst DGJ, Stieg DC, Kiss ZAC, Hanley SE, Ganesan V (2019). Cyclin C: The Story of a Non-Cycling Cyclin. Biology (Basel).

[46] Wang K, Yan R, Cooper KF, Strich R (2015). Cyclin C mediates stress-induced mitochondrial fission and apoptosis. Mol Biol Cell.

[47] Stieg DC, Willis SD, Ganesan V, Ong KL, Scuorzo J, Song M (2018). A complex molecular switch directs stress-induced cyclin C nuclear release through SCF(Grr1)-mediated degradation of Med13. Mol Biol Cell.

[48] Willis SD, Stieg DC, Ong KL, Shah R, Strich AK, Grose JH (2018). Snf1 cooperates with the CWI MAPK pathway to mediate the degradation of Med13 following oxidative stress. Microb Cell.

[49] Ito M, Yuan CX, Malik S, Gu W, Fondell JD, Yamamura S (1999). Identity between TRAP and SMCC complexes indicates novel pathways for the function of nuclear receptors and diverse mammalian activators. Mol Cell.

[50] Napoli C, Schiano C, Soricelli A (2019). Increasing evidence of pathogenic role of the Mediator (MED) complex in the development of cardiovascular diseases. Biochimie.

[51] Yamamoto T, Shimojima K, Ondo Y, Shimakawa S, Okamoto N (2017). MED13L haploinsufficiency syndrome: A de novo frameshift and recurrent intragenic deletions due to parental mosaicism. Am J Med Genet A.

[52] Lum-Naihe K, Toedebusch R, Mahmood A, Bajwa J, Carmack T, Kumar SA (2017). Cardiovascular disease progression in female Zucker Diabetic Fatty rats occurs via unique mechanisms compared to males. Sci Rep.

[53] Huss JM, Kelly DP (2004). Nuclear receptor signaling and cardiac energetics. Circ Res.

[54] Fondell JD, Ge H, Roeder RG (1996). Ligand induction of a transcriptionally active thyroid hormone receptor coactivator complex. Proc Natl Acad Sci U S A.

[55] Jones JR, Barrick C, Kim KA, Lindner J, Blondeau B, Fujimoto Y (2005). Deletion of PPARgamma in adipose tissues of mice protects against high fat diet-induced obesity and insulin resistance. Proc Natl Acad Sci U S A.

[56] Yang F, Vought BW, Satterlee JS, Walker AK, Jim Sun ZY, Watts JL (2006). An ARC/Mediator subunit required for SREBP control of cholesterol and lipid homeostasis. Nature.

[57] Zoncu R, Efeyan A, Sabatini DM (2011). mTOR: from growth signal integration to cancer, diabetes and ageing. Nat Rev Mol Cell Biol.

[58] Boutouja F, Stiehm CM, Platta HW (2019). mTOR: A Cellular Regulator Interface in Health and Disease. Cells.

[59] Gul R, Demarco VG, Sowers JR, Whaley-Connell A, Pulakat L (2012). Regulation of Overnutrition-Induced Cardiac Inflammatory Mechanisms. Cardiorenal Med.

[60] Gul R, Mahmood A, Luck C, Lum-Naihe K, Alfadda AA, Speth RC (2015). Regulation of cardiac miR-208a, an inducer of obesity, by rapamycin and nebivolol. Obesity (Silver Spring, Md).

[61] Callis TE, Pandya K, Seok HY, Tang RH, Tatsuguchi M, Huang ZP (2009). MicroRNA-208a is a regulator of cardiac hypertrophy and conduction in mice. J Clin Invest.

[62] Mann DL (2007). MicroRNAs and the failing heart. N Eng J Med.

[63] van Rooij E, Sutherland LB, Qi X, Richardson JA, Hill J, Olson EN (2007). Control of stress-dependent cardiac growth and gene expression by a microRNA. Science.

[64] Fernandes T, Barretti DL, Phillips MI, Menezes Oliveira E (2018). Exercise training prevents obesity-associated disorders: Role of miRNA-208a and MED13. Mol Cell Endocrinol.

[65] Oishi Y, Manabe I, Tobe K, Ohsugi M, Kubota T, Fujiu K (2008). SUMOylation of Krüppel-like transcription factor 5 acts as a molecular switch in transcriptional programs of lipid metabolism involving PPAR-delta. Nat Med.

[66] Drosatos K, Pollak NM, Pol CJ, Ntziachristos P, Willecke F, Valenti MC (2016). Cardiac Myocyte KLF5 Regulates Ppara Expression and Cardiac Function. Circ Res.

[67] Hsieh PN, Fan L, Sweet DR, Jain MK (2019). The Kruppel-Like Factors and Control of Energy Homeostasis. Endocr Rev.

[68] Pol CJ, Pollak NM, Jurczak MJ, Zacharia E, Karagiannides I, Kyriazis ID (2019). Cardiac myocyte KLF5 regulates body weight via alteration of cardiac FGF21. Biochim Biophys Acta Mol Basis Dis.

[69] Klein I, Danzi S (2007). Thyroid disease and the heart. Circulation.

[70] Dillmann W (2010). Cardiac hypertrophy and thyroid hormone signaling. Heart Fail Rev.

[71] Klein I, Danzi S (2016). Thyroid Disease and the Heart. Curr Probl Cardiol.

[72] Gencer B, Collet TH, Virgini V, Bauer DC, Gussekloo J, Cappola AR (2012). Subclinical thyroid dysfunction and the risk of heart failure events: an individual participant data analysis from 6 prospective cohorts. Circulation.

[73] Mitchell JE, Hellkamp AS, Mark DB, Anderson J, Johnson GW, Poole JE (2013). Thyroid function in heart failure and impact on mortality. JACC Heart Fail.

[74] Tang YD, Kuzman JA, Said S, Anderson BE, Wang X, Gerdes AM (2005). Low thyroid function leads to cardiac atrophy with chamber dilatation, impaired myocardial blood flow, loss of arterioles, and severe systolic dysfunction. Circulation.

[75] Morkin E (2000). Control of cardiac myosin heavy chain gene expression. Microsco Res Tech.

[76] Bluhm WF, Meyer M, Sayen MR, Swanson EA, Dillmann WH (1999). Overexpression of sarcoplasmic reticulum Ca(2+)-ATPase improves cardiac contractile function in hypothyroid mice. Cardiovasc Res.

[77] Vetter R, Rehfeld U, Reissfelder C, Fechner H, Seppet E, Kreutz R (2011). Decreased cardiac SERCA2 expression, SR Ca uptake, and contractile function in hypothyroidism are attenuated in SERCA2 overexpressing transgenic rats. Am J Physiol Heart Circ Physiol.

[78] Samuel TJ, Rosenberry RP, Lee S, Pan Z (2018). Correcting Calcium Dysregulation in Chronic Heart Failure Using SERCA2a Gene Therapy. Int J Mol Sci.

[79] Zhihao L, Jingyu N, Lan L, Michael S, Rui G, Xiyun B (2020). SERCA2a: a key protein in the Ca(2+) cycle of the heart failure. Heart Fail Rev.

[80] Ito M, Roeder RG (2001). The TRAP/SMCC/Mediator complex and thyroid hormone receptor function. Trends Endocrinol Metab.

[81] Pavri R, Lewis B, Kim TK, Dilworth FJ, Erdjument-Bromage H, Tempst P (2005). PARP-1 determines specificity in a retinoid signaling pathway via direct modulation of mediator. Mol Cell.

[82] Minerath RA, Dewey CM, Hall DD, Grueter CE (2019). Regulation of cardiac transcription by thyroid hormone and Med13. J Mol Cell Cardiol.

[83] Wang Z, Yang Y, Xiong W, Zhou R, Song N, Liu L (2020). Dexmedetomidine protects H9C2 against hypoxia/reoxygenation injury through miR-208b-3p/Med13/Wnt signaling pathway axis. Biomed Pharmacother.

[84] Paulin R, Sutendra G, Gurtu V, Dromparis P, Haromy A, Provencher S (2015). A miR-208-Mef2 axis drives the decompensation of right ventricular function in pulmonary hypertension. Circ Res.

[85] Castillo-Armengol J, Fajas L, Lopez-Mejia IC (2019). Inter-organ communication: a gatekeeper for metabolic health. EMBO Rep.

[86] Lee JH, Bassel-Duby R, Olson EN (2014). Heart- and muscle-derived signaling system dependent on MED13 and Wingless controls obesity in Drosophila. Proc Natl Acad Sci U S A.

[87] Nakamura M, Sadoshima J (2014). Heart over mind: metabolic control of white adipose tissue and liver. EMBO Mol Med.

[88] Baskin KK, Grueter CE, Kusminski CM, Holland WL, Bookout AL, Satapati S (2014). MED13-dependent signaling from the heart confers leanness by enhancing metabolism in adipose tissue and liver. EMBO Mol Med.

[89] Amoasii L, Holland W, Sanchez-Ortiz E, Baskin KK, Pearson M, Burgess SC (2016). A MED13-dependent skeletal muscle gene program controls systemic glucose homeostasis and hepatic metabolism. Genes Dev.

[90] Amoasii L, Sanchez-Ortiz E, Fujikawa T, Elmquist JK, Bassel-Duby R, Olson EN (2019). NURR1 activation in skeletal muscle controls systemic energy homeostasis. Proc Natl Acad Sci U S A.

[91] Amoasii L, Olson EN, Bassel-Duby R (2018). Control of Muscle Metabolism by the Mediator Complex. Cold Spring Harb Perspect Med.

[92] Cinti S (2018). Adipose Organ Development and Remodeling. Compr Physiol.

[93] Hong SK, Haldin CE, Lawson ND, Weinstein BM, Dawid IB, Hukriede NA (2005). The zebrafish kohtalo/trap230 gene is required for the development of the brain, neural crest, and pronephric kidney. Proc Natl Acad Sci U S A.

[94] Rau MJ, Fischer S, Neumann CJ (2006). Zebrafish Trap230/Med12 is required as a coactivator for Sox9-dependent neural crest, cartilage and ear development. Dev Biol.

[95] Wang X, Yang N, Uno E, Roeder RG, Guo S (2006). A subunit of the mediator complex regulates vertebrate neuronal development. Proc Natl Acad Sci U S A.

[96] Zhang H, Emmons SW (2000). A C. elegans mediator protein confers regulatory selectivity on lineage-specific expression of a transcription factor gene. Genes Dev.

[97] Janody F, Martirosyan Z, Benlali A, Treisman JE (2003). Two subunits of the Drosophila mediator complex act together to control cell affinity. Development.

[98] Segert J, Schneider I, Berger IM, Rottbauer W, Just S (2018). Mediator complex subunit Med12 regulates cardiac jelly development and AV valve formation in zebrafish. Prog Biophys Mol Biol.

[99] Rocha PP, Scholze M, Bleiss W, Schrewe H (2010). Med12 is essential for early mouse development and for canonical Wnt and Wnt/PCP signaling. Development.

[100] Tutter AV, Kowalski MP, Baltus GA, Iourgenko V, Labow M, Li E (2009). Role for Med12 in regulation of Nanog and Nanog target genes. J Biol Chem.

[101] Rienzo M, Casamassimi A, Schiano C, Grimaldi V, Infante T, Napoli C (2012). Distinct alternative splicing patterns of mediator subunit genes during endothelial progenitor cell differentiation. Biochimie.

[102] Clark RD, Graham JM Jr, Friez MJ, Hoo JJ, Jones KL, McKeown C (2009). FG syndrome, an X-linked multiple congenital anomaly syndrome: the clinical phenotype and an algorithm for diagnostic testing. Genet Med.

[103] Lujan JE, Carlin ME, Lubs HA (1984). A form of X-linked mental retardation with marfanoid habitus. Am J Med Genet.

[104] Isidor B, Lefebvre T, Le Vaillant C, Caillaud G, Faivre L, Jossic F (2014). Blepharophimosis, short humeri, developmental delay and hirschsprung disease: expanding the phenotypic spectrum of MED12 mutations. Am J Med Genet A.

[105] Graham JM Jr, Visootsak J, Dykens E, Huddleston L, Clark RD, Jones KL (2008). Behavior of 10 patients with FG syndrome (Opitz-Kaveggia syndrome) and the p.R961W mutation in the MED12 gene. Am J Med Genet A.

[106] Amodeo S, Vitrano G, Guardino M, Paci G, Corselli F, Antona V (2020). What is the impact of a novel MED12 variant on syndromic conotruncal heart defects? Analysis of case report on two male sibs. Ital J Pediatr.

[107] Ito J, Sono T, Tasaka M, Furutani M (2011). MACCHI-BOU 2 is required for early embryo patterning and cotyledon organogenesis in Arabidopsis. Plant Cell Physiol.

